# Moderate Heat Challenge Increased Yolk Steroid Hormones and Shaped Offspring Growth and Behavior in Chickens

**DOI:** 10.1371/journal.pone.0057670

**Published:** 2013-02-22

**Authors:** Aline Bertin, Marine Chanson, Joël Delaveau, Frédéric Mercerand, Erich Möstl, Ludovic Calandreau, Cécile Arnould, Christine Leterrier, Anne Collin

**Affiliations:** 1 Institut National de la Recherche Agronomique (INRA), UMR85, Physiologie de la Reproduction et des Comportements, Nouzilly, France; 2 CNRS, UMR7247, Nouzilly, France; 3 Université François Rabelais de Tours, Tours, France; 4 IFCE, Nouzilly, France; 5 INRA, UE1295, PEAT, Nouzilly, France; 6 Department of Biomedical Sciences, Medical Biochemistry, University of Veterinary Medicine, Vienna, Austria; 7 INRA, UR83 Recherches Avicoles, Nouzilly, France; Federal University of Parana (UFPR)–Campus Palotina, Brazil

## Abstract

**Background:**

Environmental challenges might affect the maternal organism and indirectly affect the later ontogeny of the progeny. We investigated the cross-generation impact of a moderate heat challenge in chickens. We hypothesized that a warm temperature–within the thermotolerance range- would affect the hormonal environment provided to embryos by mothers, and in turn, affect the morphology and behavioral phenotype of offspring.

**Methodology/Principal Findings:**

Laying hens were raised under a standard thermal condition at 21°C (controls) or 30°C (experimental) for 5 consecutive weeks. A significant increase was observed in the internal temperature of hens exposed to the warm treatment; however plasma corticosterone levels remained unaffected. The laying rate was not affected, but experimental hens laid lighter eggs than the controls during the treatment. As expected, the maternal thermal environment affected yolk hormone contents. Eggs laid by the experimental hens showed significantly higher concentrations of yolk progesterone, testosterone, and estradiol. All chicks were raised under standard thermal conditions. The quality of hatchlings, growth, feeding behavior and emotional reactivity of chicks were analyzed. Offspring of experimental hens (C30 chicks) were lighter but obtained better morphological quality scores at hatching than the controls (C21 chicks). C30 chicks expressed lesser distress calls when exposed to a novel food. Unlike C21 chicks, C30 chicks expressed no preference for energetic food.

**Conclusion/Significance:**

Our findings suggest that moderate heat challenge triggers maternal effects and modulate the developmental trajectory of offspring in a way that may be adaptive. This suggests that the impact of heat challenges on captive or wild populations might have a cross-generation effect.

## Introduction

Predicting species responses to environmental challenges has become a major worldwide concern. In particular, rapid changes in climatic conditions and habitat suitability have been largely recognized as the two major threats to biodiversity [1]. Animals need to adjust their behavior and physiology in order to cope with the changes in environmental conditions. In addition to this immediate influence, the exposure of maternal organisms to warm temperature could engender maternal effects and cause a major proportion of variance in morphological and behavioral traits of offspring. Maternal heat stress could, for example, affect the wing size and shape in the offspring of *Drosophila* [2]. In mammals like farm animals or rodents, maternal heat stress impairs reproductive functions and reduces fetal growth [3], brain weight, and learning ability [4]. Environmental maternal effects seem to be ubiquitous across a large range of taxa and broadly refer to the modification of offspring phenotype as a consequence of maternal phenotypic responses to environmental challenges or, non-genetic inheritance [5].

Not only wild populations, but also animals raised in captivity are exposed to climatic changes. Heat challenge is one of the main problems in modern aviculture. The chicken is the most abundant bird species on the earth and, because of its economic importance; its immediate physiological and behavioral adaptations to heat stress are probably the most extensively studied in the avian literature [6], [7]. An increase in the body temperature and circulating levels of corticosterone, along with a decrease in the levels of thyroid hormone triodothyronine (T3), are commonly noted in chicken exposed to heat stress [8], [9]. High temperature also reduced egg production and egg physical quality; this was because of a direct effect of heat on ovarian function rather than a consequences of reduced feed intake [10]. Both wild and domesticated fowl can select the quality of their diet in order to cope with their physiological needs. In addition, reduction in food intake and a preference for low-energy diet is noted in fowls exposed to warm temperatures [11], [12]. Many studies, on a large range of avian species, have shown that yolk hormone levels vary according to the quality of the maternal environment. Yolk hormones are known to play a key role in the mediation of maternal effects and regulation of offspring development and behaviors [13]. Breeding density [14], [15], frequency of social intrusions or social instability [16], [17], food abundance [18], parasite exposure [19], and maternal social status [20] or male attractiveness [21] were identified as environmental sources that caused variations in yolk hormone levels. However, to our knowledge, the consequences of maternal thermal environment on offspring development and behavior have not yet been explored.

The domestic hen is particularly suitable for the investigation of the potential maternal effects induced by the thermal environment since the thermotolerance limits of this bird are well described. In addition, precocial chicks can feed by themselves, allowing us the distinction between the influences of the experience *in ovo* from that of the post-hatching parental behavior. In the Japanese quail (*Coturnix c. japonica*), a close-related species, social instability, human-animal relationship, unpredictable stress, and housing conditions of females [17], [22], [23], [24] were identified as factors that influence egg quality and yolk hormone levels (androgens, progesterone). Variations in these factors were consistently found to affect offspring development and behavior. More specifically, there was a concomitant increase or decrease in the emotional reactivity of chicks according to the maternal prevailing environment. This impact of maternal effects on fearfulness was also confirmed by experimental elevation of yolk testosterone levels [25], [26]. Selection on fearfulness was also found to have correlated effects on yolk steroids content [27]. Very few studies have investigated the effects of maternal environment in domestic hens. Housing conditions [28], unpredictable access to food [29], [30], and maternal social status [20] cause variations in yolk hormone levels in hens. This caused, modifications in growth, feeding behaviors, and emotional reactivity in chicks [28], [29], [30].

In this study, whether an elevated temperature—within the range of thermotolerance—would cause modifications in yolk hormones levels and modify the phenotype of the offspring in laying hens was investigated. For this, laying hens were maintained at either standard temperature conditions (21°C) or 30°C for 5 consecutive weeks. All chicks were raised under standard temperature conditions. The egg quality, hatchling quality, and chick behavior were analyzed using via tonic immobility test, open-field test, food choice test and novel food test. We predict that adaptation of hens to warm temperature would trigger maternal effects. We expected modifications in egg quality, including yolk hormones levels, and subsequent modification in offspring development and behavior. We focused on emotional reactivity and feeding behavior of chicks since large inter-individual variations are commonly observed in these traits, and these could strongly affect the ability of animals to cope with different types of farming conditions. The mechanisms underlying yolk hormone deposition are still under investigation [31] and ‘natural’ maternal challenges were found to increase or decrease yolk hormone levels. Therefore, predicting the way in which warm temperature affects yolk steroid levels and offspring phenotype remains difficult. This pioneering study showed that moderate heat challenge triggers maternal effects and affects both the maternal population and subsequent phenotype of offspring.

## Materials and Methods

### Ethics statement

All birds were maintained at the Experimental Unit PEAT of INRA (Nouzilly, France). The Experimental Unit is registered by the ministry of Agriculture with the license number B-37-175-1 for animal experimentation. All experiments were approved by the Ethic Committee in Animal Experimentation of Val de Loire CEEA Vdl (permit number 2011-02-8). The CEEA vdl is registered by the National Committe ‘Comité National de Réflexion Ethique sur l'Expérimentation Animale’ under the number 19. All experiments were performed in accordance with the European Communities Council Directive 2010/63/UE.

### Laying hens

#### Housing conditions and treatment

Forty 20-week old White leghorn hens (*Gallus gallus domesticus*) from the experimental unit PEAT (INRA, Nouzilly) were equally stratified into 2 groups. The groups were balanced for the hen mass and egg mass. The hens of the 2 groups were housed in 2 similar thermo-regulated rooms (40 m^2^). Each bird was placed in an individual wire home-pen (100×100×50 cm) (length × width × height) containing wood shavings on the floor, a nest, perch, drinker, and trough. In each room, birds had tactile, visual, and vocal contacts with the others. Water and food (calculated content: ME = 2800 kcal/kg; crude protein = 16%; lysine = 0.7%; calcium = 0.4%) were available *ad libitum* during a 14:10 h light:dark cycle. All the birds were maintained at a temperature of 21.0±0.5°C for a period of 2 weeks. After this period, the room temperature of the experimental group was gradually elevated to 30.0±0.5°C over 3 days. This temperature was maintained for the following 5 weeks. This temperature is considered as a moderate heat challenge for White leghorn hens. These birds had a physiological potential to maintain their laying rate under 30°C. Detrimental effects are commonly known to appear with temperatures above 32°C [32]. The control group was maintained at a temperature of 21±0.5°C (standard breeding temperature) throughout the experiment.

#### Morpho-physiological measures on hens

Each hen was weighted 5 times: once a week before the beginning of the treatment and once every week during the first 4 weeks of treatment. Rectal temperature of each bird was recorded twice: once before the treatment and once after 3 weeks of treatment; the temperature was measured using a digital thermometer (Testo, Forbach, France). The daily feed intake was measured once before the treatment and once during the first 3 weeks of treatment. The weight of each trough was measured after every 24 h to determine the daily intake of each hen.

Plasma corticosterone levels were measured to evaluate the impact of the heat treatment on hens. Blood samples were collected twice for each hen: once the day before the beginning of the treatment and once after 3 weeks of treatment. Hen's wing was held motionless for 30 seconds in order to sample blood from the wing vein. At each sampling time, 2 mL of blood was obtained, collected in tubes containing EDTA (2 mg/mL) and kept on ice. For both sampling days, all animals were sampled between 9:30 and 11:00 am. The blood samples were then centrifuged at 2,000× g for 15 min at 4°C. The plasma was collected and stored at −20°C before the measurement at the Biological Center of Chizé (France). Corticosterone was extracted by adding 3 mL diethyl-ether to 100 µL of each plasma sample, followed by vortexing and centrifugation. The diethyl-ether phase containing the steroid was decanted and discarded after snap freezing the tube in an alcohol bath at 38°C. The organic solvent was then evaporated. The dried extracts were redissolved in 300 µL of phosphate buffer, and corticosterone concentration was assayed in duplicate. Next, 100 µL of the extract was incubated overnight with 5,000 cpm of the appropriate 3H-steroid (Perkin Elmer, US) and polyclonal rabbit corticosterone-21-thyroglobulin antiserum (Sigma, US). The bound fraction was then separated from the free fraction by adding dextran-coated charcoal and radioactivity of the antibody bound fraction was measured using a tri-carb 2810 TR scintillation counter (Perkin Elmer, US). Tests were performed to validate the corticosterone assay on plasma. Inter- and intra-assay variations were respectively 9.99% and 7.07%. The lowest detectable concentration of corticosterone was 0.14 ng/mL. Two samples were serially diluted in the assay buffer, and their displacement curves were parallel to the standard curve. The mean recovery of the standard spiked in a sample was 92%.

#### Tonic Immobility

Since heat stress was shown to increase the tonic immobility duration in laying hens [33], which could indicate a state of stress in animals, all the hens were tested once before the heat period and after 3 weeks of treatment. This test followed a procedure similar to that described by Jones [34]. Animals were caught individually and carried to another room. Each hen was then placed on its back in a U-shaped wooden cradle and held by the experimenter with one hand over the sternum and one gently covering the bird's head. Each hen was restrained for 10 s and then released. When more than 10 s passed between release and the bird's escape, duration of tonic immobility was measured. If not, another induction attempt was conducted. A score of zero second was given when tonic immobility could not be attained after 5 induction attempts. The test was stopped, and a maximum duration of 300 s was allocated when the hen did not stand up within 300 s. The observer remained out of the hen's sight during the test. Duration of tonic immobility and number of induction attempts were recorded. The duration of the tonic immobility reaction is considered to be a standard and robust measure of fearfulness [34]. This manipulation induces a reversible catatonic state, the duration of which is positively correlated with the general underlying fearfulness [35], [36].

#### Laying rate

Eggs from all females were collected daily for 6 consecutive weeks; i.e., 1 week before the heat period and during the 5-week treatment. The laying rate was calculated as the number of laid eggs/female/day.

### Eggs

#### Incubation and yolk hormonal assays

Since the formation of individual yolks requires 21 days and the hens were progressively acclimated to heat, egg collection for incubation and hormonal assays was started on the 21^st^ day after the beginning of the heat period [37]. Eggs were collected during 15 days in each group and stored at 17°C for incubation. The hens were fertilized by artificial insemination on the fifth and sixth days before egg collection and then once a week during two weeks. To that end, each hen was gently maintained approximately 30 s and 50 µL of a pure mixture of sperms, originating from 10 males, was deposited at the entrance of the cloacal vent with a pipette. Each female contributed on an average 4.27±0.36 eggs, and a total of 270 eggs were collected. Of the 270 eggs collected, 171 were fertile and maintained in the incubator (n = 90 control eggs; n = 81 experimental eggs). All eggs were placed in alternative rows on each shelf of the incubator. They were maintained at 37.8°C and 56% relative humidity and turned automatically and continuously. At day 14 of incubation, the eggs containing dead embryos were eliminated. Three days before hatching, the rotation was stopped, and the temperature was decreased to 37.6°C. Eggs were then placed in a grid constructed of a wire mesh and cardboard dividers so that chicks from both the sets could be identified.

During the collection period, 2 eggs per female were used for hormonal assays and stored at −20°C. Eggs sampled for hormonal assay were weighted, and the frozen yolk was separated from the albumen. Eggshells were separated, dried for 24 h, and weighed. The weight of the albumen was determined by subtracting the weight of the eggshell and the yolk from that of the whole egg. The 2 eggs per female were used to determine the mean egg weight and mean relative proportion of each component (yolk, albumen, shell) for each female. Although most research on yolk hormones has focused on the effects of testosterone levels [38], concentrations of yolk progesterone and androstenedione might also play important roles in precocial birds [22], [39]. In addition, the light/dark cycle or housing conditions were found to modulate yolk estradiol levels in laying hens [28], [30]. Therefore, whether yolk testosterone, androstenedione, progesterone, and estradiol levels were modulated by maternal environment was investigated. The presence of corticosterone in egg yolk remains controversial [40] and was not found to be modulated by environmental conditions in laying hens [29], [30] nonetheless its potential implications were also investigated to provide a more complete picture of yolk content. One yolk sample per female was analyzed for corticosterone and estradiol concentrations (Biological Center of Chizé, France). Another yolk per female was analyzed for measuring immunoreactive progesterone, androstenedione, and testosterone concentrations (Veterinary University of Vienna, Austria).

#### Yolk corticosterone and estradiol radio-immunoassays

Yolk concentrations of corticosterone and estradiol were assayed using the same procedure. Briefly, 100 mg of each sample was homogenised in 1 mL of distilled water. Steroids were extracted by adding 3 mL of diethyl-ether to 300 µL of the mixture, followed by vortexing and centrifugation. The diethyl-ether phase containing steroids was decanted and discarded after snap freezing the tube in an alcohol bath at 38°C. This procedure was repeated twice for each yolk, and the organic solvents were then evaporated. The extracts were redissolved in 600 µL of phosphate buffer, and each hormone was assayed in duplicate. Next, 100 µL of the extract was incubated overnight with 5,000 cpm of the appropriate _3_H-steroid (Perkin Elmer, US) and polyclonal rabbit antiserum. Anti-11-HS-corticosterone antiserum was supplied by P.A.R.I.S. (France) and anti-estradiol by Sigma (US). The bound fraction was then separated from the free fraction by adding dextran-coated charcoal, and the activity was counted on a tri-carb 2810 TR scintillation counter (Perkin Elmer, US). Tests were performed to validate both the hormone assays on egg yolk samples. Inter-assay and intra-assay variations for corticosterone and estradiol were 20.98% and 16.70 and 17.18% and 13.13% respectively. The lowest detectable concentrations of corticosterone and estradiol were 1.55 pg/mg, and 0.69 pg/mg respectively. Two yolk samples were serially diluted in the assay buffer, and their displacement curves were found to be parallel to the standard curve. The mean recoveries of the standard spiked sample for corticosterone and estradiol were 120% and 90% respectively.

#### Yolk progesterone, testosterone, and androstenedione assays

The concentrations of progesterone (P4) and androgens (androstenedione = A4 and testosterone = T) were assayed using a method similar to that described by Lipar et al. [41], Möstl et al. [42] and Hackl et al. [43]. Each yolk was cut in half. Because the distribution of hormones vary between egg layers [41], [42], the mixed half yolk was assayed. After thawing the yolk was mixed and 0.5 g of each yolk was diluted with 1.5 ml of water and vortexed for 30 sec. The emulsion was diluted with 8 ml methanol, vortexed for 30 min and stored overnight at minus 20 °C. After centrifugation (minus 10 °C 1500 g, 15 min) the supernatant was diluted with assay buffer (1+10) and used for enzyme immunoassays (EIAs) for measuring the concentrations of immunoreactive progesterone, testosterone and androstenedione [44]. Inter-assay coefficients of variation were 12.2%, 10.7% and 10.1% respectively. The intra-assay variation was 8.5%, 4.2% and 9.2%.

### Chicks

#### Morpho-physiological measures

We kept 96 chicks (48 controls and 48 experimental), all hatched on the 21^st^ day of incubation for the experiment. Each chick was identified with a numbered ring on its leg. The chicks were weighted, and their body temperature under the wing was recorded using an infrared thermometer (TES1326S). The temperature was also recorded at 6 days of age.

Approximately 2 h after the chicks hatched, their quality was assessed by 2 experimenters. Of the 8 parameters completely described by Tona et al. [44], 6 were used in this study. Each chick received a quality score that varie from 0 (bad quality) to 40 (good quality) (Table 1). The chicks were weighed at hatching, and at 6, 13 and 20 days.

**Table 1:** Allocation of scores to morphological parameter observations (completely described by Tona et al. [41]).

Parameters	Characteristics	Scores
Eyes	opened	2
	closed	0
Activity	good	2
	weak	0
Legs	normal	8
	1 twisted leg	4
	2 twisted legs	0
Navel	closed and flat	12
	swollen and red	4
	presence of a scab	4
	swollen and red with a scab	0
Remaining membrane	no membrane	8
	small membrane	4
	large membrane	0
Remaining yolk	no yolk	8
	small yolk	4
	large yolk	0

Between 17 and 25 weeks of age, female offspring were weighted and placed in individual cages to record the onset of laying and laying rate.

#### Housing conditions

The 96 chicks were placed in pairs in plastic tubs measuring 50×40×40 cm with a wire top and a floor covered by wood shavings; the chicks were divided in 2 groups. Each group consisted of 24 pairs of chicks: 24 pairs of control chicks (C21) and 24 pairs of experimental chicks (C30). The pairs of chicks were randomly allocated to 2 rooms of equal size and maintained on a 10-h:14-h light:dark cycle; water was provided ad libitum. Chicks were fed *ad libitum* by using a conventional starter mash (Experimental Unit PEAT, INRA centre de Tours, France). The food was dispensed in 50-cm-long feeding troughs. The troughs were covered with a metallic roof having 12 circular holes (diameter, 5 cm); these holes allowed sufficient access for the chicks to the feed while avoiding food spillage. Two opaque drinking bottles (1 L) with pipettes were placed in each cage. Ambient temperature was maintained at 33.0±1.5 °C from hatching until the chicks were 8-days-old; subsequently, it was decreased progressively to 24±1 °C, until the chicks were 25-days-old. The sex of the animals was determined by observation of the comb and legs at 3 weeks of age. The control and experimental groups consisted of 26 females and 22 males, and 21 females and 27 males, respectively.

#### Analysis of feeding behavior

The daily intake was measured for each pair of chicks at 3, 11, and 17 days. The weight of each trough was recorded every 24 h to determine the food consumption by each pair.

Food preference: the preference of chicks for their standard food (fat content: 6.6%) or an energetic food (mash insectivore; La Ferme de Manon, France, fat content: 13%) was tested when they were 10 days old. For four consecutive days before the test, all the pairs of chicks were exposed to 50 g of the energetic mash placed in a cup in addition to their regular food in order to acclimate them to the food to allow them to assimilate the nutritional value of the mash [45]. Because chicks are extremely distressed when isolated, the study was conducted in the pairs of chicks (n = 24 pairs per treatment). The test box, identical to the home box, was located in a different room. Each pair of chicks was placed in the test box after 1 h of food deprivation. They were transported in a 15×15×15 cm container, deposited in the center of the test box, and observed for 5 min. The test box contained 2 troughs identical to their familiar trough; 1 containing 100 g of the familiar food and another containing 100 g of the energetic food. The location of the troughs was balanced across the test trials. An observer (blind to the treatment), hidden behind a curtain with small observation windows, recorded the behavior of 1 focal bird of each pair. Focal birds were chosen randomly beforehand and identified by a colored mark on the head. The experimenter recorded the latencies to explore each type of food (the bird touch the food with its beack), the latencies to ingest each type of food and the time spent eating on each trough (the bird was considered be eating when the movements of the mandible, neck, and throat due to swallowing were observed). Because the food particles weigh very less, the quantity of food eaten could not be weighed with precision over such a short period.

The behavior of chicks toward energetic item was also tested using a choice test between a control solution (600 mL water) and a solution (600 mL water) containing 5% sucrose placed in the home cage. A concentration of 5% sucrose is detectable by chicks and do not engender a preference or an avoidance compared to tap water [46]. The localization of the drinking bottle containing the sweet solution was counterbalanced between the cages. The liquid intake was recorded for each pair by weighing the bottles on a 24-h scale when the chicks were 21–22 days of age. Two pairs of C30 chicks and 3 pairs of C21 chicks drank exclusively from 1 out the 2 drinkers and were excluded from the analysis.

Food neophobia: food neophobia (i.e. fear of novel foods) is particularly well described in birds [47] and can be influenced by maternal effects [48]. Food neophobia in offspring and habituation to a novel food were investigated using a protocol described by Bertin et al., [49]. Chicks were exposed twice to a novel food; once at 12 days of age and once at 19 days of age. After 1 h of food deprivation, pairs of chicks were exposed for 3 min to a familiar trough containing 100 g of millet seeds following the same protocol as the food preference test. An experimenter recorded the latency to ingest the novel food and the number of distress calls.

#### Analysis of the emotional reactivity

A tonic immobility test, as described for adult hens, was performed on all chicks when they were 7 days of age. The number of inductions and the tonic immobility duration were recorded.

Open field test: the open-field test involves transferring the birds from a familiar home cage to an unfamiliar and open environment. Chicks were individually placed in the middle of an open cylindrical arena (diameter, 120 cm; height, 35 cm) on a linoleum floor for 5 min. To assess their locomotor activity, 2 perpendicular lines were drawn in the arena, dividing it into 4 equal parts. The latency of the first step, number of distress calls, and activity of the birds (number of times when a bird crossed a line) were recorded by an experimenter hidden behind a curtain with observation windows. Animal activity in an open-field test is considered inversely correlated with emotional reactivity. Quiet and inactive animals are commonly considered to have a higher level of emotional reactivity than active animals [50]. This test was performed when the chicks were 23–24 days of age.

### Data analysis

Kolmogorov-Smirnov tests determined whether the data were normally distributed. In the hens, all the morpho-physiological and behavioral measures were analyzed using one-way analysis of variance (ANOVA) for repeated measures (treatment × time). When required, PLSD Fisher post-hoc tests were performed. The relative proportions of egg components (yolk and eggshell) were determined and analyzed using multivariate analysis of variance (MANOVA). If Wilks' Lambda tests showed a significant multivariate effect, individual one-way ANOVAs were performed for each dependent variable. Yolk hormone concentrations were analyzed using a MANOVA and individual one-way ANOVAs. Hatching success and the number of hatchlings obtaining the maximum morphological quality score were tested using a Chi-square test. The body temperature of chicks, the laying rate of the female offspring, and feed intake were tested using ANOVA for repeated measures with the treatment and time as factors. The mass of chicks was analyzed using ANOVA for repeated measures with the treatment and sex as factors. In the food choice test, a chi-square test was conducted on the number of animals choosing to first ingest the energetic food. T-tests were conducted within each group on the other variables (the same for the choice test with water). Inter-group comparisons were analyzed using ANOVA on latency scores (latency to eat the energetic food–latency to eat the standard food), and the total time spent eating during testing. The number of distress calls and the latencies to ingest in the novel food test were analyzed using ANOVA for repeated measures with treatment and sex as factors. Variables recorded in the tonic immobility and open-field tests were analyzed using ANOVAs. Data are presented as mean ± SEM. All analyses were performed using Statview software (SAS, Cary, NC), with significance accepted at P≤0.05.

## Results

### Impact of the heat treatment on adult hens

#### Morpho-physiological parameters

**Figure 1:**
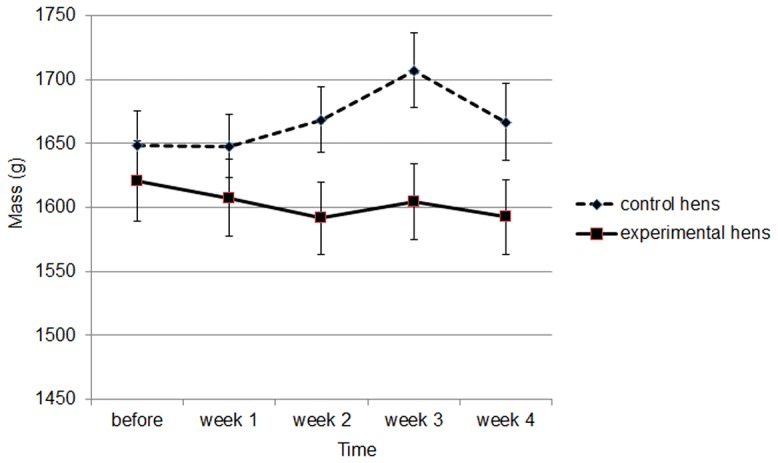
Mean ± SEM mass of control and experimental hens before and during the treatment.

The mass of hens did not differ significantly between the 2 groups (ANOVAR, F_1,38_ = 2.68, *P* = 0.10) (Fig. 1). There was a significant effect of time (ANOVAR, F_4,38_ = 4.2, *P* = 0.002) and a significant interaction between time and treatment (ANOVAR, F_4,152_ = 7.2, *P*<0.001). Within experimental hens we observed an effect of weeks. They were significantly lighter than before the heat period at weeks 2 and 3 (*t*-test, *P*<0.05 for all comparisons). An effect of weeks was also observed in controls hens which were significantly heavier than before at week 2 (*t*-test, *t* = 3.2, *P* = 0.004).

**Figure 2:**
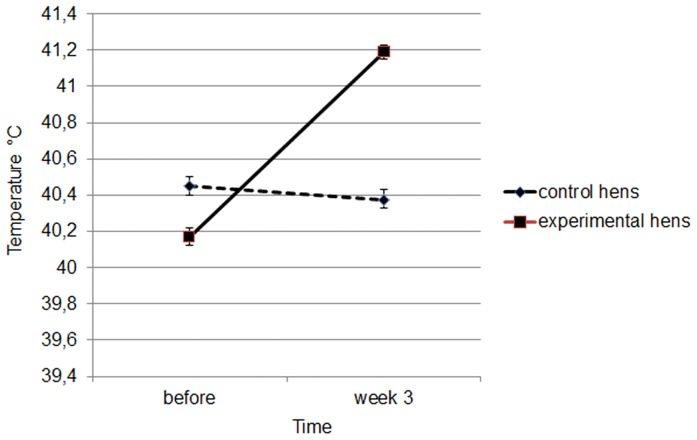
Mean ± SEM internal temperature of control and experimental hens before and during the treatment.

The treatment had a significant effect on the internal temperature of hens (ANOVAR, F_1,38_ = 24.49, *P*<0.001). There was a significant effect of time (ANOVAR, F_1,38_ = 89.23, *P*<0.001) and a significant interaction between time and treatment (ANOVAR, F_1,38_ = 119.98, *P*<0.001). The internal temperature of the experimental group increased (Fig. 2) and was significantly higher than that of the controls after 3 weeks of treatment (PLSD post-hoc Fisher test, *P*<0.001).

The treatment had no significant effect on plasma corticosterone concentrations (corticosterone concentrations before the treatment: controls vs. experimental hens: 1.67±0.17 ng/mL vs. 1.52±0.12 ng/mL; during the heat treatment: 1.52±0.12 vs. 1.52±0.14; ANOVAR, treatment effect, F_1,38_ = 0.13, *P* = 0.72; time effect, F_1,38_ = 2.75, *P* = 0.1; time × treatment, F_1,38_ = 0.17, *P* = 0.67).

The treatment did not have a significant effect on the daily feed intake. Irrespective of the treatment, the feed intake increased during the course of the experiment (before the treatment, controls vs. experimental hens: 108.1±10.5 g vs. 110.3±6.9 g; week 1: 166.1±19.8 g vs. 137.6±19.4 g; week 2: 142±19.4 g vs. 125±10.6 g; week 3: 104.4±16.1 g vs. 149.1±24.1 g, ANOVAR, treatment effect, F_1,38_ = 0.17, *P* = 0.68; time effect, F_3,38_ = 3.44, *P* = 0.02; treatment × time, F_3,114_ = 0.96, *P* = 0.41).

#### Tonic immobility tests

The treatment had no significant effect on tonic immobility duration or the number of induction attempts (duration before the treatment, control vs. experimental hens: 154.85±31.55 s vs. 105.6±20.75 s; during the treatment: 109.2±20.01 s vs. 92.9±25.87 s; ANOVAR, treatment effect, F_1,38_ = 1.68, *P* = 0.20; time effect, F_1,38_ = 1.39, *P* =  0.24; treatment × time, F_1,38_ = 0.44, *P* = 0.51; number of inductions before the treatment, controls vs. experimental hens: 0.7±0.2 vs. 0.7±0.2; during the treatment: 1.1±0.4 vs. 0.9±0.3; ANOVAR, treatment effect, F_1,38_ = 0.12, *P* = 0.72; time effect, F_1,38_ = 1.45, *P* = 0.23; treatment × time, F_1,38_ = 0.16, *P* = 0.69).

#### Egg laying

The heat treatment had no significant effect on the laying rate (mean number of egg/female/day before the treatment, control vs. experimental hens: 0.75±0.04 vs. 0.76±0.03; week 1: 0.93±0.02 vs. 0.86±0.03; week 2: 0.83±0.04 vs. 0.81±0.03; week 3: 0.9±0.02 vs. 0.86±0.03; week 4: 0.8±0.02 vs. 0.82±0.03; ANOVAR, treatment effect, F_1,38_ = 0.56, *P* = 0.46; time effect, F_4,38_ = 7.75, *P*<0.001; treatment × time, F_4,152_ = 0.74, *P* = 0.56). The laying rate increased over time, irrespective of the treatment.

#### Egg quality and yolk hormones

The heat treatment had a significant effect on the mass of eggs: 50.75±0.56 g for control vs. 48.16±0.73 g for the experimental hens (ANOVA, F_1,38_ = 8, *P* = 0.007). However, there was no overall effect on the egg component masses (yolk/egg weight: control vs. experimental hens: 0.26±0.003 g vs. 0.25±0.004 g; albumen/egg weight: 0.65±0.004 g vs. 0.65±0.004 g; eggshell/egg weight: 0.093±0.002 g vs. 0.092±0.002; MANOVA, F_3,36_ = 1.47, *P* = 0.23).

**Figure 3:**
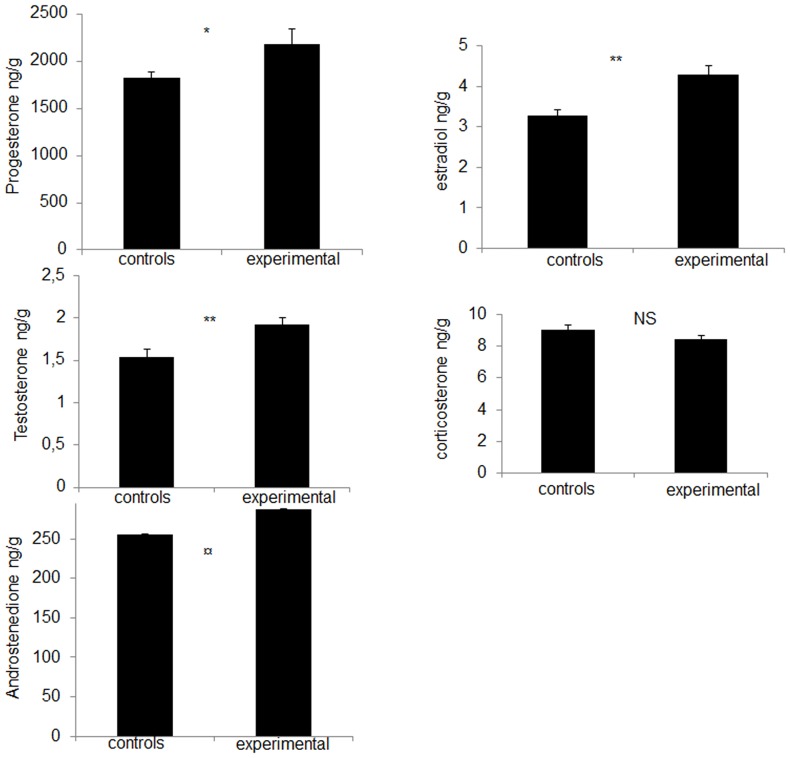
Yolk steroid levels in the eggs from control (N = 20) and experimental hens (N = 20). Mean ± SEM progesterone (P4), testosterone (T), androstenedione (A4), estradiol and corticosterone concentrations (ng/g of yolk) in the eggs of laying hens maintained under a standard thermal condition (21°C) or under a warm temperature (30°C) for 5 weeks. ANOVAs, ^*^ *P*<0.05; ^**^ *P*<0.01; ¤ 0.05<*P*<0.1; NS, *P*>0.05.

The treatment had an overall significant effect on yolk hormone concentrations (MANOVA, F_5,34_ = 6.63, *P*<0.01). The eggs from experimental hens contained significantly higher concentrations of progesterone, testosterone and estradiol (ANOVAs, *P*<0.05) (Fig. 3). Eggs of experimental hens also tended to have higher concentrations of androstenedione (ANOVA, F_1,38_ = 2.97, *P* = 0.09). No significant differences between groups were observed in yolk corticosterone concentrations (ANOVA, F_1,38_ = 2.47, *P* = 0.12) (Fig. 3).

### Characterization of the offspring

#### Morpho-physiological measures

**Figure 4:**
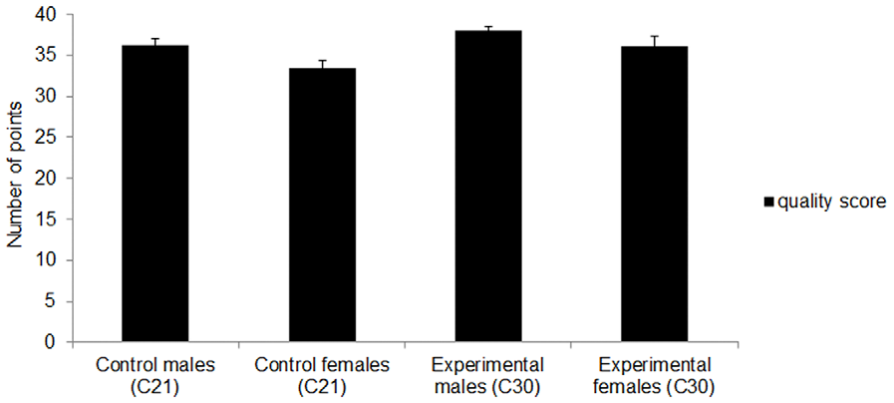
Mean ± SEM quality scores of C21 and C30 hatchlings (C21 male chicks: N = 21; C21 female chicks: N = 26; C30 male chicks: N = 27; C30 female chicks: N = 21).

The number of chicks hatched did not differ significantly between the 2 groups (Table 2, Chi2, *P*>0.05). The quality score was significantly higher in the chicks from the experimental hens (C30) than in the control chicks (C21) (Fig. 4; ANOVA, F_1,91_ = 5.71, *P* = 0.02). In addition, a higher number of C30 chicks obtained the maximum score compared to the controls: 29 chicks vs. 17 chicks (Chi2; *P*<0.05). A significant sex effect with no interaction between sex and treatment were observed (sex effect, F_1,91_ = 6.21, *P* = 0.01; sex × treatment, F_1,91_ = 0.22, *P* = 0.63). Irrespective of the treatment, male chicks had higher quality scores than females (PLSD Fisher test, *P* = 0.006).

**Table 2:** Number of fertile eggs and hatching success in control and experimental hens.

Treatment	number of fertile eggs	number of embryonic deaths	number of eggs hatched
Control (21°C)	90	10	77
Experimental (30°C)	81	11	67

The body temperature did not differ significantly between C30 and C21 chicks at hatching or at 6 days of age (body temperature at hatching, C30 vs. C21 chicks: 37.2±0.14 °C vs. 37.2±0.13°C; at 6 days of age: 36.3±0.16°C vs. 36.5±0.16°C; ANOVAR, treatment effect, F_1,94_ = 0.25, *P* = 0.62, time effect, F_1,94_ = 27.43, *P*<0.001, treatment × time, F_1,94_ = 1.52, *P* = 0.22). For both the groups, the temperature decreased between hatching and 6 days of age.

There was a significant effect of the treatment on the mass of chicks. C30 chicks were significantly lighter than C21 chicks until 20 days of age (Table 3) (ANOVAR, treatment effect, F_1,94_ = 9.97, *P* = 0.002). A significant sex effect with no interaction between sex and treatment was observed (sex effect, F_1,94_ = 10.67, *P* = 0.001; sex × treatment, F_1,94_ = 0.18, *P* = 0.66). Irrespective of the treatment, male chicks were bigger than females from 6 to 20 days of age (PLSD Fisher test, *P*<0.05).

**Table 3:** Mean ± SEM mass of C30 and C21 chicks from days 1 to 20.

	Weight			
Chicks	day 1	day 6	day 13	day 20
Control (C21)	36.9±0.4^b^	52.9±0.6^b^	102.9±1.7^b^	196.9±2.6^b^
Experimental (C30)	34.7±0.3^a^	50.4±0.7^a^	99.7±1.4^a^	189.8±2.5^a^

Different letters indicate significant differences in post-hoc Fisher tests.

Female offspring were kept to record the onset of egg laying. At 17 weeks of age, the mass of females did not differ significantly between the groups (C21 females, n = 25, mass = 1257.12±22.34 g and C30 females, n = 21, mass = 1218.09±24.19 g; ANOVA, F_1.44_ = 1.4, *P* = 0.24). Between 17 and 25 weeks of age, the laying rate of the female offspring did not differ significantly between the treatments (mean number of eggs/female/day at 17 weeks of age in C30 females: 0.05±0.02 and 0.81±0.07 and at 25 weeks of age; in C21 females: 0.12±0.05 and 0.76±0.05; ANOVAR, treatment effect, F_1,45_ = 0.01, *P* = 0.9; time effect, F_1,45_ = 193, *P*<0.001; treatment × time, F_1,45_ = 1.69, *P* = 0.2).

#### Analysis of feeding behavior

The daily feed intake did not differ significantly between C30 and C21 chicks (C30 vs. C21 chicks: day 3: 22.04±1.54 g vs. 22.29±1.83 g; day 10: 50.33±3.14 g vs. 52.37±3.55 g; day 17: 60.79±3.25 g vs. 58.62±2.69 g; ANOVAR, treatment effect, F_1,46_ = 0.0004, *P* = 0.98, time effect, F_2,46_ = 98.15, *P*<0.001, treatment × time, F_2,92_ = 0.28, *P* = 0.75).

Significantly lesser C30 chicks chose the energetic food first than C21 chicks (8 chicks out 48 vs. 17 chicks out 48, chi2 = 5.34, *P* = 0.02). Within the control group, the latency to ingest the energetic food was significantly shorter that to ingest the standard food (52.7±16 s vs. 148.6±23.6 s, *t*-test, *t* = 3.26, *P* = 0.003) whereas the latencies to ingest each type of food did not differ significantly within the C30 group (110.1±22.8 s vs. 59.9±16.3 s, *t*-test, *P* = 0.12). The latency scores revealed that C30 chicks had significantly longer latencies to approach the energetic food than the controls (−50.25±31.55 s vs. 95.92±29.35 s, ANOVA, F_1,46_ = 11.5, *P* = 0.001). The total time spent eating during testing did not differ significantly between the groups (C30 vs. C21 chicks: 55.58±5.02 s vs. 54.29±8.76, ANOVA, F_1,46_ = 0.01, *P* = 0.89).

In the choice test between water and sweet water, no preference was observed within the control group (54.52±4.68 g vs. 45.52±6.32 g, *t*-test, *t* = 1.12, *P* = 0.27). However, the C30 chicks showed a higher water intake than for the solution of sucrose (61.18±4.55 g vs. 40.9±3.93 g, *t*-test, *t* = 2.49, P = 0.02).

**Figure 5:**
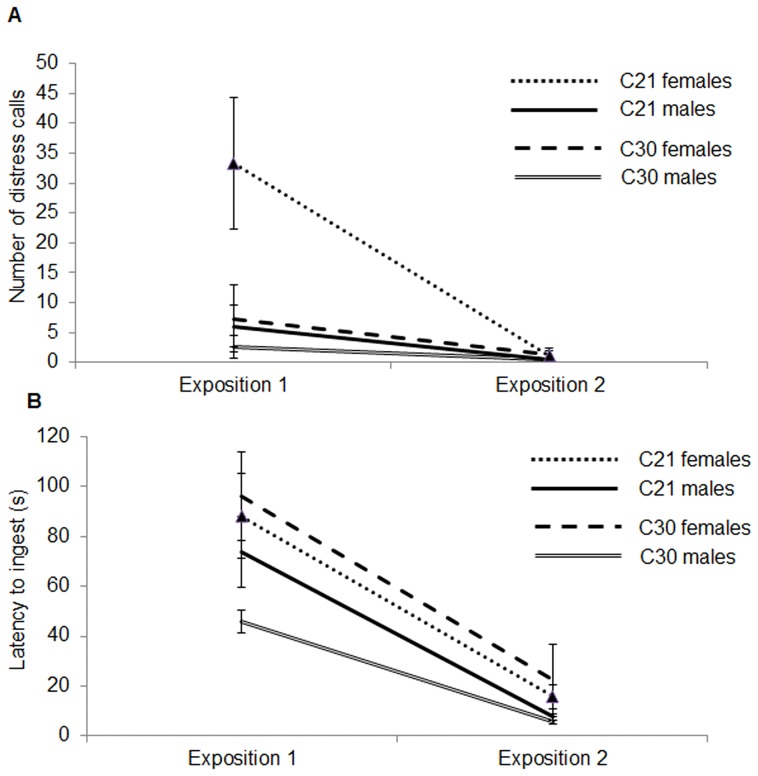
Mean ± SEM number of distress calls (a) and latency to ingest the novel food (b) during the first and second expositions to the novel food (C21 male chicks: N = 21; C21 female chicks: N = 26; C30 male chicks: N = 27; C30 female chicks: N = 21).

In the novel food test, the number of distress calls emitted differed significantly between the groups and between sex (Fig. 5A) (ANOVAR, treatment effect, F_1,44_ = 4.37, *P* = 0.04; sex effect, F_1,44_ = 5.71, *P* = 0.02; treatment × sex, F_1,44_ = 2.57, *P* = 0.11). Irrespective of the treatment, male chicks expressed significantly lesser distress calls than female chicks. A bigger decrease in the number of distress calls is observed in C21 chicks compared to C30 chicks which expressed few distress calls during the first exposition (treatment × time, F_1,44_ = 5.94, *P* = 0.02). The latency to ingest the novel food did not differ significantly between the 2 groups (Fig. 5B) (ANOVAR, treatment effect, F_1,44_ = 0.17, P = 0.68; sex effect, F_1,44_ = 5.59, *P* = 0.02; treatment × sex, F_1,44_ = 0.17, *P* = 0.25). Irrespective of the treatment, male chicks ingested the novel food faster than the female chicks.

#### Analysis of the emotional reactivity

The number of inductions and duration of tonic immobility did not differ significantly between the 2 groups (number of inductions: C30 chicks vs. C21 chicks, 2.06±0.3 vs. 2.3±0.30, ANOVA, F_1,94_ = 5.59, *P* = 0.4; duration: 29.15±5.25 s vs. 23.39±4.42, ANOVA, F_1,94_ = 0.66, *P* = 0.41).

No significant differences were observed between the groups in the open-field test (latency of the first step: C30 chicks vs. C21 chicks, 56.69±5.28 s vs. 58.81±6.41 s; ANOVA, F_1,94_ = 0.06, *P* = 0.79; number of lines crossed: 3.25±0.32 vs. 3.42±0.43, ANOVA, F_1,94_ = 5.59, *P* = 0.76; number of distress calls: 119.67±8.4 vs. 99.17±8.5, ANOVA, F_1,94_ = 2.94, *P* = 0.09).

## Discussion

The results of the present study contribute to the knowledge of non-genetic maternal influences on development and phenotype outcome of offspring. The body temperature and yolk steroid hormone levels were increased in the group of hens exposed to a warm temperature of 30°C. Besides yolk testosterone concentration—the target of most studies investigating maternal effects in birds—yolk progesterone and estradiol concentrations were also affected. The offspring of the females exposed to heat showed a better morphological quality score at hatching but light weight. In addition, unlike control chicks, chicks from females exposed to heat were less distressed when exposed to a novel food and showed no attraction for energetic food or drink. Exposure to warm temperatures could modify the egg resources provided by mothers, like egg size and yolk hormones, causing a transgenerational influence on the ontogeny of the offspring.

Hens exposed to heat showed a significant increase in the internal temperature as well as a slight decrease in the body mass. However, there was no significant effect on the feed intake, laying rate, plasma corticosterone levels, or tonic immobility duration. The plasma corticosterone levels observed correspond to basal levels observed in standard conditions [51]. One of the immediate responses to heat challenge is the development of hyperthermia. Hyperthermia enables sufficient heat loss by radiation, convection, and conduction and enables the maintenance of the body temperature within a relatively narrow range [7]. The lack of a significant effect on the plasma corticosterone level or tonic immobility duration indicates that the physiological adaptation to the experimental temperature probably did not activate the hypothalamic-pituitary-adrenal (HPA) axis which suggest that the treatment did not engendered a state of ‘chronic stress’ in experimental hens. Corticosterone levels could have increased initially and normalized later, but our study findings are in accordance with those reported by previous studies showing that plasma corticosterone levels are not necessarily affected by mild heat exposure in laying hens [52]. Our results on tonic immobility duration also indicate that a difference in the underlying fearfulness between control and experimental hens was not likely to explain the differences in yolk hormone concentrations.

Eggs from the experimental hens were lighter, but the relative proportion of egg components was found to be not affected. A reduction in egg mass with no interruption in laying rate is commonly observed in laying hens exposed to mild heat [53]. This reduction in egg mass is thought to be the result of a decrease in feed intake, ovary weight, and body mass [10], [54], [55]. Controlling food spillage in adult hens maintained on floor is very difficult and, hence, reduction in feed intake could not be totally excluded despite our non-significant measures.

In addition to this modification of egg size, remarkable differences were observed in yolk hormone concentrations. Eggs of experimental hens contained significantly higher levels of yolk progesterone, testosterone and estradiol. Yolk androstenedione concentration was almost significantly higher in the experimental hens, whereas yolk corticosterone concentration was unaffected. Progesterone is the precursor of androgens and estrogens and is produced in the granulosa cells of the pre-ovary follicles [56]. In quail, hens and other precocial bird species, considerably higher amounts of progesterone than those of T or A4 were found around the germinal disc (outer layer of the yolk) [43]. One possible explanation for the higher level of progesterone observed in the experimental eggs could be the higher production of the follicular wall. The conversion of progesterone by side-chain cleavage could have resulted in the higher levels of androgens and estrogens. Exposing laying hens to acute heat stress (12 h daily to 42±3°C) was found to engender a significant reduction in the mRNA expression of 2 steroidogenic enzymes (17β-estradiol and P450 17-α hydroxylase) [10]. If this was thought to be the underlying mechanism in our study, the yolk androgen or estradiol levels would have been expected to be lower in the eggs of experimental hens.

A growing body of literature shows that maternal stress during egg formation—acute stress or stress mimicked with subcutaneous corticosterone implantation in hens—increase plasma corticosterone levels and decrease the synthesis of reproductive hormones which accumulate in the yolk [57], [58], [59]. However, there was no evidence of an increase in plasma or yolk corticosterone in the present study. In addition, the levels of androgens and immunoreactive progesterone were clearly higher in the eggs of experimental hens. Mild environmental challenges do not necessarily engender the activation of the HPA axis in birds. For example, quail exposed to mild chronic stress for 1 week did not show any change in their basal corticosterone levels at the end of the stress period [60]. Guibert et al. [23] exposed quail to unpredictable stressors and found an increased yolk testosterone level. Similarly, Nätt et al. [30] reported an increase in the level of reproductive hormone (estradiol) in the eggs of hens exposed to environmental challenges (unpredictable diurnal light rhythm). Our data suggest that the maternal environment can influence the production of yolk hormones via other pathways than the HPA axis.

Depending on the type and duration of the challenges, the regulatory mechanism for the production of yolk hormones might be at the level of the ovary or include circulating hormones other than glucocorticoids. The diminished reproductive performance in hens subjected to heat stress is thought to be, in part, related to an increased secretion of prolactine, which is produced by the pituitary gland [61]. Circulating prolactine concentrations are known to reduce follicular steroidogenesis in fowl [62]; however, surprisingly, in American kestrels (*Falco sparverius*), an increase in plasma prolactin concentrations was found to elevate yolk-testosterone concentrations both in laboratory and field conditions [63]. The Circulating prolactine might thus have contributed to the increase of yolk steroids observed in experimental eggs. The mechanisms of how environmental conditions during egg laying translate into variations in yolk steroid concentrations remain to be elucidated. To our knowledge, this is the first study to analyze environmental influence on yolk hormones from progesterone (precursor of androgens and corticosterone) to estradiol (at the end of the metabolic chain). The present findings will help targeting potential biosynthesis pathways, key steps in advancing the understanding of maternal effects.

Heat stress had no significant effect on hatching duration or hatching success; however, despite a lower body mass, the quality scores of C30 hatchlings were higher than those of C21 hatchlings. In addition, a higher number of C30 hatchlings obtained the maximum quality score. Day-old chicks are the end-product of the hatchery industry, and good-quality 1-day-old chicks are a crucial link between hatchery and farmers. Since even minor navel conditions could be associated with a reduction in the production efficiency of broilers [64], only chicks of good quality (normal conformation of legs, good activity, completely sealed and clean navels, no yolk sacs or dried membranes protruding from the navel area) are considered as marketable and constitute the starting material for farmers. Day old quality was found to be related to several factors such as incubator quality, incubation environment, age of breeders and egg storage conditions [44]. Our data showed that the thermal environment provided to laying hens could also affect the quality of chicks. In passerine birds, exposure to maternal androgens correlates positively with the developmental rate of embryos [65]. One possible hypothesis could be that the higher concentration of androgens in the eggs of experimental hens accelerated the development and maturation of embryos and in turn advance, for example, the stage of navel healing at hatching. Yolk hormones were consistently found to enhance or impair the growth of chicks and commonly have a short-duration effect on the post-hatching development [13], [17], [22], [23], [24] however, the smaller size of our experimental chicks was most likely because of the smaller size of the experimental eggs. In fact, heavier eggs are known to produce larger chicks [66], [67]. The absence of a difference in the mass and laying performance of the female offspring suggests a catch-up growth during the development.

There were no significant differences between C30 and C21 chicks in the tonic immobility and open-field tests. The tonic immobility response is a fear reaction to physical restraint. This reaction is considered as the last one in a series of defensive behaviors displayed by a bird in response to attack by a predator [34]. The open-field test is commonly used in precocial birds to assess fear of novel and open environments. This test also assesses the response of precocial young birds to separation from flockmates [50]. Our results suggested that the maternal thermal environment might have no influence on the general underlying fearfulness or social reinstatement behavior of the progeny. However, Janczak et al. [29] reported higher duration of tonic immobility in offspring of hens exposed to an unpredictable feed restriction treatment (despite no difference in yolk hormone contents). In addition, in the precocial quail, variation in yolk hormonal contents was almost consistently found to influence the responses in open-field (particularly the locomotor activity) and/or tonic immobility tests [17], [23], [24], [26], [27], [68], [69]. Hence, concluding that the quality of egg, because of the thermal maternal environment, had no influence on such behavioral traits would be too early. Hegyi and Schwabl [39] have argued that different yolk androgens interact and differently affect offspring phenotypes. For example, the authors reported that an injection of A4 in the yolk increased the locomotor activity in the open-field test but had no effect on the mass of quail chicks, whereas T injection reduced mass gain and had no effect on the open-field responses. Our data showed that the maternal thermal environment induced variation in both egg size and yolk steroids concentrations. All the components of the ‘ontogenetic niche’ provided to the embryos probably interact and influence different dimensions of fear-related behaviors.

Such an effect on different dimensions of fear-related behaviors was highlighted in our study by the clear difference in the frequency of distress calls expressed when subjected to a novel food. Latencies to ingest food did not differ, but the lower number of distress calls in C30 than in C21 indicated a lower fear response in the former chicks upon first encountering the food. Maternal effects on food neophobia remained overlooked in vertebrates despite the description of this fear response across a wild range of taxa. In zebra finches (*Taeniopygia guttata*), increase in yolk T-level has shown to facilitate habituation to a novel food. In addition, as in our study, males were found to be faster than females to approach novel foods [48]. Taken together, these results indicated that maternal effects probably shape the later ability of birds to adapt to novel food resources. Understanding the influence of maternal effects on food neophobia is of considerable interest in both captive and wild populations of birds. In poultry farming, animals are exposed to changes in the composition of their diet during the course of their development. When facing these changes, animals can express strong distress responses that dramatically impairs animal welfare [70], [71]. Although further investigations in this regard are necessary, slightly enhancing the maternal thermal environment of parental population could be advantageous to obtain hatchlings with better quality scores and reduced neophobia. Many experimental studies have focused on determining ways to deactivate food neophobia [47] and so far, its determinism remained unknown. Our data might facilitate a novel line of research investigating how the maternal environment triggers maternal effects on food neophobia and the adaptability of populations. Although extrapolating our results to wild populations is very speculative, indicating recent hypothesis of considering food neophobia as the emotion that determines the capacity of wild animals to innovate and adapt to the changes in their environmental conditions might be of interest [72], [73].

Our data also showed differences in the feeding behavior of the progeny of experimental hens. In the food choice test, more C21 birds than C30 birds chose the energetic food first. In addition, C21 chicks were faster to ingest the energetic food compared to the standard food whereas C30 expressed no preference. In the choice test between water and sweet water, C30 chicks ingested more tap water than sweet water whereas C21 chicks showed no preference. Our data suggest maternal effects on feeding preferences of chicks. This influence of the maternal environment on the behavior of the offspring toward more or less energetic items was already reported by Nätt et al. [30]. Offspring of hens exposed to unpredictable food access showed higher preference for sunflower seeds (high energy food) than for low energy pellets. Chicks from hens exposed to this chronic stress treatment were also exposed *in ovo* to higher yolk estradiol levels and were bigger than control chicks. These data and our results suggest that the quality of the prenatal environment probably influence growth and the metabolism of animals. Precocial birds commonly learn from their parents which food to eat [74] but they also taste and eat by themselves different food items few hours after hatching. According to the quality of their prenatal environment, young animals might thus orient spontaneously their pecking toward less or more energetic food that could be advantageous in a warm environment. However, as the egg already contains around 60,000 cells by the time the egg is laid, we could no totally rule out that the observed effects on the development and behavior of chicks could be due to an early exposure to a higher temperature in the experimental group.

In conclusion, our results further the knowledge that environmental challenges might play an important role in rapid adaptations across generations. Our study revealed complex mechanisms of maternal effects including both egg mass and yolk hormones. Although the mechanisms and adaptive functions remain to be investigated, our results suggest that the thermal environment of bird populations during laying might have the potential to engender phenotypic variability in subsequent generations. Whether epigenetic mechanisms are implicated remains to be elucidated. These findings suggest a new perspective on non-genetic inheritance and adaptation to climatic challenges.

## References

[1] Barnagaud J-Y, Devictor V, Jiguet F, Barbet-Massin M, Le Viol I, et al (2012) Relating Habitat and Climatic Niches in Birds. PLoS ONE 7: e32819. 22427891 10.1371/journal.pone.0032819 PMC3299694

[2] Andersen DH, Pertoldi C, Scali V, Loeschcke V (2005) Heat stress and age induced maternal effects on wing size and shape in parthenogenetic *Drosophila mercatorum* . J Evol Biol 18: 884–892. 16033560 10.1111/j.1420-9101.2005.00955.x

[3] Hansen PJ (2009) Effects of heat stress on mammalian reproduction. Philosophical Transactions of the Royal Society B: Biological Sciences 364: 3341–3350. 10.1098/rstb.2009.0131 PMC2781849 19833646

[4] Jonson KM, Lyle JG, Edwards MJ, Penny RHC (1976) Effect of prenatal heat stress on brain growth and serial discrimination reversal learning in the guinea pig. Brain Res Bull 1: 133–150. 974792 10.1016/0361-9230(76)90056-3

[5] Harper LV (2005) Epigenetic inheritance and the intergenerational transfer of experience. Psychol Bull 131: 340–360. 15869332 10.1037/0033-2909.131.3.340

[6] Renaudeau D, Collin A, Yahav S, de Basilio V, Gourdine JL, et al (2012) Adaptation to hot climate and strategies to alleviate heat stress in livestock production. Animal 6: 707–728. 22558920 10.1017/S1751731111002448

[7] Yahav S (2009) Alleviating heat stress in domestic fowl: different strategies. World's Poult Sci J 65: 719–732.

[8] Iqbal A, Decuypere E, Elazim AA, Kuhn ER (1990) Prehatch and posthatch high-temperature exposure affects the thyroid-hormones and corticosterone response to acute heat-stress in growing chicken (Gallus-domesticus). J Therm Biol 15: 149–153.

[9] Yahav S (2000) Domestic fowl - Strategies to confront environmental conditions. Avian Poultry Biol Rev 11: 81–95.

[10] Rozenboim I, Tako E, Gal-Garber O, Proudman JA, Uni Z (2007) The Effect of Heat Stress on Ovarian Function of Laying Hens. Poult Sci 86: 1760–1765. 17626822 10.1093/ps/86.8.1760

[11] MacLeod MG, Dabutha LA (1997) Diet selection by Japanese quail (Coturnix coturnix japonica) in relation to ambient temperature and metabolic rate. Br Poult Sci 38: 586–589. 9511005 10.1080/00071669708418040

[12] Yo T, Siegel PB, Faure JM, Picard M (1998) Self-selection of dietary protein and energy by broilers grown under a tropical climate: Adaptation when exposed to choice feeding at different ages. Poult Sci 77: 502–508. 9565230 10.1093/ps/77.4.502

[13] Groothuis TGG, Muller W, von Engelhardt N, Carere C, Eising C (2005) Maternal hormones as a tool to adjust offspring phenotype in avian species. Neurosci Biobehav Rev 29: 329–352. 15811503 10.1016/j.neubiorev.2004.12.002

[14] Schwabl H (1997) The contents of maternal testosterone in house sparrow Passer domesticus eggs vary with breeding conditions. Naturwissenschaften 84: 406–408. 9353760 10.1007/s001140050418

[15] Groothuis TG, Schwabl H (2002) Determinants of within- and among-clutch variation in levels of maternal hormones in Black-Headed Gull eggs. Funct Ecol 16: 281–289.

[16] Mazuc J, Bonneaud C, Chastel O, Sorci G (2003) Social environment affects female and egg testosterone levels in the house sparrow (Passer domesticus). Ecol Lett 6: 1084–1090.

[17] Guibert F, Richard-Yris M-A, Lumineau S, Kotrschal K, Guémené D, et al (2010) Social Instability in Laying Quail: Consequences on Yolk Steroids and Offspring's Phenotype. PLoS ONE 5: e14069. 21124926 10.1371/journal.pone.0014069 PMC2989911

[18] Verboven N, Monaghan P, Evans DM, Schwabl H, Evans N, et al (2003) Maternal condition, yolk androgens and offspring performance: a supplemental feeding experiment in the lesser black-backed gull (Larus fuscus). Proc R Soc Lond Ser B-Biol Sci 270: 2223–2232. 10.1098/rspb.2003.2496 PMC1691499 14613608

[19] Vedder O, Kingma SA, von Engelhardt N, Korsten P, Groothuis TGG, et al (2007) Conspecific brood parasitism and egg quality in blue tits Cyanistes caeruleus. J Avian Biol 38: 625–629.

[20] Muller W, Eising CM, Dijkstra C, Groothuis TGG (2002) Sex differences in yolk hormones depend on maternal social status in Leghorn chickens (Gallus gallus domesticus). Proc R Soc Lond Ser B-Biol Sci 269: 2249–2255. 10.1098/rspb.2002.2159 PMC1691150 12427318

[21] Schwabl H (1993) Yolk is a source of maternal testosterone for developing birds. Proc Natl Acad Sci U S A 90: 11446–11450. 8265571 10.1073/pnas.90.24.11446 PMC48000

[22] Bertin A, Richard-Yris MA, Houdelier C, Lumineau S, Mostl E, et al (2008) Habituation to humans affects yolk steroid levels and offspring phenotype in quail. Horm Behav 54: 396–402. 18572170 10.1016/j.yhbeh.2008.04.012

[23] Guibert F, Richard-Yris M-A, Lumineau S, Kotrschal K, Bertin A, et al (2011) Unpredictable mild stressors on laying females influence the composition of Japanese quail eggs and offspring's phenotype. Appl Anim Behav Sci 132: 51–60.

[24] Guesdon V, Bertin A, Houdelier C, Lumineau S, Formanek L, et al (2011) A Place to Hide in the Home-Cage Decreases Yolk Androgen Levels and Offspring Emotional Reactivity in Japanese Quail. PLoS ONE 6: e23941. 21980338 10.1371/journal.pone.0023941 PMC3182999

[25] Bertin A, Richard-Yris M-A, Möstl E, Lickliter R (2009) Increased yolk testosterone facilitates prenatal perceptual learning in Northern bobwhite quail (Colinus virginianus). Horm Behav 56: 416–422. 19646986 10.1016/j.yhbeh.2009.07.008

[26] Daisley JN, Bromundt V, Mostl E, Kotrschal K (2005) Enhanced yolk testosterone influences behavioral phenotype independent of sex in Japanese quail chicks *Coturnix japonica* . Horm Behav 47: 185–194. 15664022 10.1016/j.yhbeh.2004.09.006

[27] Bertin A, Richard-Yris MA, Houdelier C, Richard S, Lumineau S, et al (2009) Divergent selection for inherent fearfulness leads to divergent yolk steroid levels in quail. Behaviour 146: 757–770.

[28] Janczak AM, Torjesen P, Rettenbacher S (2009) Environmental effects on steroid hormone concentrations in laying hens' eggs. Acta Agricultura Scandinavica Section A, Animal Science 59: 80–84.

[29] Janczak AM, Torjesen P, Palme R, Bakken M (2007) Effects of stress in hens on the behaviour of their offspring. Appl Anim Behav Sci 107: 66–77.

[30] Nätt D, Lindqvist N, Stranneheim H, Lundeberg J, Torjesen PA, et al (2009) Inheritance of Acquired Behaviour Adaptations and Brain Gene Expression in Chickens. PLoS ONE 4: e6405. 19636381 10.1371/journal.pone.0006405 PMC2713434

[31] Groothuis TGG, Schwabl H (2008) Hormone-mediated maternal effects in birds: mechanisms matter but what do we know of them? Philos Trans R Soc B-Biol Sci 363: 1647–1661. 10.1098/rstb.2007.0007 PMC2606725 18048291

[32] Arieli A, Meltzer A, Berman A (1980) The thermoneutral temperature zone and seasonal acclimatization in the hen. Br Poult Sci 21: 471–478. 7260693 10.1080/00071668008416699

[33] Campo JL, Carnicer C (1994) Effects of several stressors on tonic immobility reaction of chickens. Arch Geflugelkd 58: 75–78.

[34] Jones RB (1986) The tonic immobility reaction of the domestic-fowl - a review. World's Poult Sci J 42: 82–96.

[35] Jones RB, Mills AD, Faure JM (1991) Genetic and experiential manipulation of fear-related behavior in Japanese quail chicks (*Coturnix coturnix japonica*). J Comp Psychol 105: 15–24. 2032451 10.1037/0735-7036.105.1.15

[36] Mills AD, Faure JM (1991) Divergent selection for duration of tonic immobility and social reinstatement behavior in Japanese quail (Coturnix coturnix japonica) chicks. J Comp Psychol 105: 25–38. 2032452 10.1037/0735-7036.105.1.25

[37] Sauveur B, Reviers M (1988) Reproduction des volailles et production d'oeufs: Institut national de la recherche agronomique.

[38] Pfannkuche KA, Gahr M, Weites IM, Riedstra B, Wolf C, et al (2011) Examining a pathway for hormone mediated maternal effects - Yolk testosterone affects androgen receptor expression and endogenous testosterone production in young chicks (Gallus gallus domesticus). Gen Comp Endocrinol 172: 487–493. 21536043 10.1016/j.ygcen.2011.04.014

[39] Hegyi G, Schwabl H (2010) Do different yolk androgens exert similar effects on the morphology or behaviour of Japanese quail hatchlings Coturnix japonica? J Avian Biol 41: 258–265.

[40] Rettenbacher S, Moestl E, Groothuis TGG (2009) Gestagens and glucocorticoids in chicken eggs. Gen Comp Endocrinol 164: 125–129. 19501091 10.1016/j.ygcen.2009.05.019

[41] Lipar JL, Ketterson ED, Nolan V, Casto JM (1999) Egg yolk layers vary in the concentration of steroid hormones in two avian species. Gen Comp Endocrinol 115: 220–227. 10417235 10.1006/gcen.1999.7296

[42] Mostl E, Spendier H, Kotrschal K (2001) Concentration of immunoreactive progesterone and androgens in the yolk of hens' eggs (*Gallus domesticus*). Wien Tierärztl Mschr 88: 62–65.

[43] Hackl R, Bromundt V, Daisley J, Kotrschal K, Mostl E (2003) Distribution and origin of steroid hormones in the yolk of Japanese quail eggs (*Coturnix coturnix japonica*). J Comp Physiol B-Biochem Syst Environ Physiol 173: 327–331. 10.1007/s00360-003-0339-7 12677460

[44] Tona K, Bamelis F, De Ketelaere B, Bruggeman V, Moraes VMB, et al (2003) Effects of egg storage time on spread of hatch, chick quality, and chick juvenile growth. Poult Sci 82: 736–741. 12762394 10.1093/ps/82.5.736

[45] Kyriazakis I, Tolkamp BJ, Emmans G (1999) Diet selection and animal state: an integrative framework. Proc Nutr Soc 58: 765–771. 10817142 10.1017/s0029665199001044

[46] Gentle MJ (1972) Taste preference in the chicken (Gallus domesticus L.). Br Poult Sci 13: 141–155. 5017939 10.1080/00071667208415928

[47] Marples NM, Kelly DJ (1999) Neophobia and dietary Conservatism: Two distinct processes? Evol Ecol Res 13: 641–653.

[48] Tobler M, Sandell MI (2007) Yolk testosterone modulates persistence of neophobic responses in adult zebra finches, *Taeniopygia guttata* . Horm Behav 52: 640–645. 17878055 10.1016/j.yhbeh.2007.07.016

[49] Bertin A, Calandreau L, Arnould C, Nowak R, Levy F, et al (2010) In Ovo Olfactory Experience Influences Post-hatch Feeding Behaviour in Young Chickens. Ethology 116: 1027–1037.

[50] Faure JM, Jones RB, Bessei W (1983) Fear and social motivation as factors in open-field behavior of the domestic chick - a theoretical consideration. Biol Behav 8: 103–116.

[51] Kjaer JB, Guémené D (2009) Adrenal reactivity in lines of domestic fowl selected on feather pecking behavior. Physiology & Behavior 96: 370–373. 19027766 10.1016/j.physbeh.2008.10.023

[52] Lin H, Decuypere E, Buyse J (2006) Acute heat stress induces oxidative stress in broiler chickens. Comparative Biochemistry and Physiology - Part A: Molecular & Integrative Physiology 144: 11–17. 10.1016/j.cbpa.2006.01.032 16517194

[53] Yahav S, Shinder D, Razpakovski V, Rusal M, Bar A (2000) Lack of response of laying hens to relative humidity at high ambient temperature. Br Poult Sci 41: 660–663. 11201448 10.1080/713654988

[54] Savory CJ (1986) Influence of ambient temperature on feeding activity parameters and digestive function in domestic fowls. Physiology & Behavior 38: 353–357. 3786516 10.1016/0031-9384(86)90106-x

[55] Mashaly MM, Hendricks GL III, Kalama MA, Gehad AE, Abbas AO, et al (2004) Effect of heat stress on production parameters and immune responses of commercial laying hens. Poult Sci 83: 889–894. 15206614 10.1093/ps/83.6.889

[56] Huang ES, Nalbandov AV (1979) Steroidogenesis of chicken granulosa and theca cells: in vitro incubation system. Biol Reprod 20: 442–453. 454747 10.1095/biolreprod20.3.442

[57] Henriksen R, Rettenbacher S, Groothuis TGG (2011) Prenatal stress in birds: Pathways, effects, function and perspectives. Neurosci Biobehav Rev 35: 1484–1501. 21536067 10.1016/j.neubiorev.2011.04.010

[58] Henriksen R, Groothuis TG, Rettenbacher S (2011) Elevated plasma corticosterone decreases yolk testosterone and progesterone in chickens: linking maternal stress and hormone-mediated maternal effects. PLoS ONE 6: e23824. 21886826 10.1371/journal.pone.0023824 PMC3160319

[59] Okuliarová M, Sárniková B, Rettenbacher S, Skrobánek P, Zeman M (2010) Yolk testosterone and corticosterone in hierarchical follicles and laid eggs of Japanese quail exposed to long-term restraint stress. Gen Comp Endocrinol 165: 91–96. 19524583 10.1016/j.ygcen.2009.06.007

[60] Calandreau L, Bertin A, Boissy A, Arnould C, Constantin P, et al (2011) Effect of one week of stress on emotional reactivity and learning and memory performances in Japanese quail. Behav Brain Res 217: 104–110. 20937328 10.1016/j.bbr.2010.10.004

[61] Donoghue DJ, Krueger BF, Hargis BM, Miller AM, el Halawani M (1989) Thermal stress reduces serum luteinizing hormone and bioassayable hypothalamic content of luteinizing hormone-releasing hormone in hens. Biol Reprod 41: 419–424. 2686762 10.1095/biolreprod41.3.419

[62] Zadworny D, Shimada K, Ishida H, Sato K (1989) Gonadotropin-stimulated estradiol production in small ovarian follicles of the hen is suppressed by physiological concentrations of prolactin in vitro. Gen Comp Endocrinol 74: 468–473. 2744414 10.1016/s0016-6480(89)80044-9

[63] Sockman KW, Schwabl H (2001) Plasma corticosterone in nestling American kestrels: Effects of age, handling stress, yolk androgens, and body condition. Gen Comp Endocrinol 122: 205–212. 11316426 10.1006/gcen.2001.7626

[64] Fasenko GM, O'Dea EE (2008) Evaluating broiler growth and mortality in chicks with minor navel conditions at hatching. Poult Sci 87: 594–597. 18281590 10.3382/ps.2007-00352

[65] Schwabl H, Palacios MG, Martin TE (2007) Selection for rapid embryo development correlates with embryo exposure to maternal androgens among passerine birds. Am Nat 170: 196–206. 17874371 10.1086/519397

[66] Dzialowski EM, Sotherland PR (2004) Maternal effects of egg size on emu Dromaius novaehollandiae egg composition and hatchling phenotype. J Exp Biol 207: 597–606. 14718503 10.1242/jeb.00792

[67] Ipek A, Dikmen BY (2007) The relationship between growth traits and egg weight in pheasants (P. colchicus). J Biol Environ Sci 1: 117–120.

[68] Okuliarova M, Kostal L, Zeman M (2006) Increased testosterone in egg differentially influences behaviour of Japanese quail during ontogeny. Acta Vet BRNO 325–331.

[69] Okuliarova M, Skrobanek P, Zeman M (2007) Effect of increasing yolk testosterone levels on early behaviour in Japanese quail hatchlings. Acta Vet BRNO 76: 325–331.

[70] Jones RB (1986) Responses of domestic chicks to novel food as a function of sex, strain and previous experience. Behavioural Processes 12: 261–271. 24897588 10.1016/0376-6357(86)90040-9

[71] Lecuelle S, Bouvarel I, Chagneau AM, Lescoat P, Laviron F, et al (2010) Feeding behaviour in turkeys with a change-over from crumbs to pellets. Appl Anim Behav Sci 125: 132–142.

[72] Greenberg RS (2003) The role of neophobia and neophilia in the development of innovative behavior of birds. In: Reader SM, Laland, K., editor. Animal innovation. Oxford: Oxford University Press.

[73] Sol D, Griffin AS, Bartomeus I, Boyce H (2011) Exploring or Avoiding Novel Food Resources? The Novelty Conflict in an Invasive Bird. PLoS ONE 6: e19535. 21611168 10.1371/journal.pone.0019535 PMC3097186

[74] Wauters AM, Richard-Yris MA (2002) Mutual influence of the maternal hen's food calling and feeding behavior on the behavior of her chicks. Dev Psychobiol 41: 25–36. 12115288 10.1002/dev.10042

